# Lights, camera…research! Short film and social media to recruit to HIV research in Indonesia

**DOI:** 10.1177/20552076221090049

**Published:** 2022-05-05

**Authors:** K Gedela, H Luis, RF Loverian, S Maharani, N Rajus, FS Wignall, A Garner, E Sukmaningrum, A McOwan, N Nwokolo, G Whitlock, I Irwanto

**Affiliations:** 1 9762 Chelsea and Westminster NHS Foundation Trust, London, UK; 2 558835 Yayasan Bali Peduli HIV/Sexual Health Clinic, Denpasar, Bali, Indonesia; 3 Pusat Unggulan Kebijakan Kesehatan dan Inovasi Sosial (PUI-PT PPH, PUK21S), HIV/AIDS Research Centre, 64732 Atma Jaya Catholic University, Jakarta, Indonesia; 4 Hornet Social Network, San Francisco, USA

**Keywords:** HIV, social media, film, global health research, marginalized populations

## Abstract

**Introduction:**

HIV prevalence among men who have sex with men has increased in Indonesia, amid reports of growing stigma against lesbian, gay, bisexual and transgender individuals and policies that have pushed back public health outreach to these groups.

**Methods:**

We assessed the utility of tailored short film and targeted social media engagement to recruit men who have sex with men in Indonesia to HIV social science research. A short HIV testing promotion film, anonymised short survey and invite to a wider research study was embedded on a website platform and disseminated using geo and social/community group targeting for 1 month via a social networking app and social media platforms.

**Results:**

From 3 January 2021 to 3 February 2021, there were over 2200 hits of the website within Indonesia. A total of 177 male web users who identified as men who have sex with men or preferred not to declare their sexuality, engaged by watching the short film and completing the survey, they were aged between 17 and 60 years old, of Indonesian nationality and living in Indonesia. Of these, 88% indicated having at least one HIV test in their lifetime, 66% had felt shame with respect to their sexuality and 53% indicated feeling afraid to have a HIV test. Ninety (51%) of the 177 validated using their email or mobile phone number demonstrating willingness to be contacted to join a further study. Twenty-three eligible men who have sex with men, aged 21–55 years old, joined a further social science research study. Participants were from diverse backgrounds and included men born in provinces outside Bali, of different socio-economic and employment backgrounds and diverse relationship contexts.

**Discussion:**

Engaging, empowering digital media involving key health messaging can provide health education in more effective ways, build trust and bring communities together. Targeted digital and social media approaches could reach increasingly marginalised and vulnerable communities to promote individual and public health and enable recruitment to valuable medical research.

## Introduction

Indonesia has a fast-growing HIV epidemic. HIV prevalence has increased specifically amongst men who have sex with men (MSM) from 8.5% in 2011 to 25.8% in 2018 nationally.^1,2^ However, data from 2018 suggests that in the major provinces of Jakarta and Bali, one in three MSM are reported to be living with HIV.^1,2^

HIV prevalence among MSM is reported to have increased amid reports of growing stigma and discrimination against lesbian, gay, bisexual and transgender (LGBT) individuals and policies that have pushed back public health outreach to these groups.^3–5^

In recent times, the country has observed worsening stigma and active discrimination against LGBT communities, associated with a slowing of the country's HIV response. For example, in 2019 and 2020, proposed laws that aim to criminalize sexual relations outside marriage,^4^ impacting the rights of all people and impose legal requirements for individuals and their families to report to the Government for rehabilitation if they have same-sex relationships.^5^

MSM are increasingly using social media, mobile social networking apps and other online forums to find sexual partners, but also sexual health information.^6–10^ Such platforms have shown to provide a perceived safe place to interact with other MSM, without being identified and targeted.

We assessed the utility of tailored short film and targeted social media engagement to recruit hard-to-reach, diverse, marginalised MSM communities in Indonesia to HIV social science research.

## Methods

Using audience-centred and iterative design techniques, our diverse, multi-professional and community collaboration created a 1-minute short HIV testing and health promotion film.^11^ The collaboration and iterative process has been described in a previous publication.^11^ The film featured empowering imagery alongside HIV health messaging in Bahasa Indonesia (Indonesian language). The aim of using film was to visually attract and engage Indonesian MSM living in Indonesia (targeting focused on Jakarta and Bali) to take part in the online survey and recruit to wider research involving focus group workshops. The film plus a short anonymised survey, an invite to a wider research study and a simple mobile/email validation process were embedded on a website platform (www.helpbeathiv.org); two versions of the site exist in Bahasa Indonesia and English, however, the site automatically displays in Bahasa if the user location is in Indonesia.

The survey assessed demographics for study eligibility, HIV testing behaviour and whether fear/shame were barriers to testing. The website details the aims of the research and an invite to join a wider research study involving focus group workshops to better understand risk behaviour, perceptions of stigma and the challenges MSM face in accessing HIV testing and care.

Website users who engage with the site, would choose to watch the 1 min short film first and then be able to decide whether they want to move forward with the short anonymised survey, via interactive clicks moving them through the website. Following completion of the interactive survey, the user can then decide whether to proceed, or not, to the user validation process. This allows them to express interest to know more about the wider research study and provide a valid email or mobile number. The research team can then provide more information directly to them about the wider study, forward an online participant information sheet, answer any questions and arrange a workshop date if they agree to proceed.

The website platform was disseminated using geo and social/community group targeting for 1 month between the 3 January and 3 February 2021 via the gay men's social networking app Hornet (over 25 million users worldwide) and social media platforms, Facebook (https://www.facebook.com/UTAMAIndonesia) and Instagram (@utama2021_official). Specific social media accounts were set up for this purpose and managed by the research/community teams. Paid, targeted social media boosts/promotions were applied throughout the month; five paid boosts were applied via Facebook, costing a total of £94. These posts had an estimated reach of over 63,000 people (the number of people who saw any content from the UTAMA Facebook Page or about the Page).

Targeted video posts (involving clips of the short film), promoted on Instagram gained between 1000 and 2200 views each. Due to constraints of the COVID-19 pandemic, the site was disseminated for study recruitment for only one month rather than the planned 6 months. Care was taken to use engaging imagery and social media hashtags to promote target community engagement but to avoid terms that may have sparked backlash from anti-LGBT groups.

## Results

From 3 January to 3 February 2021, there were over 2200 hits of the website within Indonesia.

During this time there were 188 individual users who watched the film and completed the online anonymised survey. Spikes in website hits and survey responses directly followed Hornet app postings and targeted promoted social media posts. After removing non-MSM, 177 male users who identified as MSM or preferred not to declare their sexuality remained. These individuals were aged between 17 and 60 years old and of Indonesian nationality and living in Indonesia. The median age was 30 years old and the interquartile range was 6 years. Of these, 88% (155 users) indicated having at least one HIV test in their lifetime (HIV status was not asked), 66% (116) had felt shame with respect to their sexuality and 53% (94) indicated feeling afraid to have a HIV test. Ninety (51%) of the 177 validated using their email address or mobile phone number demonstrating willingness to be contacted to join a further study. Living in Bali was a requirement to join the focus group workshops.

Twenty-three MSM, who were eligible, aged between 21 and 55 years old, validated to join a further research study and joined face to face, in-depth focus group workshops held in Bali in January and February. These 23 participants were web users who lived in Bali, had validated their email or mobile number to be contacted by the research team, and agreed following contact and reading the participant information sheet to take part in focus group sessions. All participants provided signed consent to take part. Ethical approval for this study was granted by the Liverpool School of Tropical Medicine, UK and from the Atma Jaya Catholic University, Jakarta, Indonesia.

All 23 men were Indonesian, MSM and the median age was 31 years (range: 21–55 years). All indicated having had at least one HIV test in their lives, however, HIV status remained anonymous. Participants were from diverse backgrounds and included men born in provinces outside Bali, of different socio-economic and employment backgrounds (e.g. agricultural workers to senior professionals), and diverse relationship contexts (e.g. married with children to open relationships with male partners). All 23 participants had completed up to primary school education in Indonesia. One participant had completed his education up to primary school level only, 39% (9 participants) had completed up to secondary or high school education, 52% (12 participants) completed a diploma or to a bachelor’s degree, and one participant had completed a master’s degree.

## Discussion

By utilising multi-professional collaborations involving community and creative groups, we believe the potential response from this form of target population engagement, involving short film, interactive website formats and social media, could be amplified to encourage greater involvement of marginalised communities in purposeful research.

We had planned to utilise appropriate social media influencers more within this process. The study recruitment period was cut from 6 months to 1 month due to limitations from the COVID-19 pandemic and a formal approach to involve appropriate social media influencers was not adopted fully. Given the known backlash to the LGBTQ community online within Indonesia, we had planned to approach and engage influencers individually and privately via their openly available contact details and via Balinese and Indonesian LGBTQ community sources, before engaging or requesting them to share our study related posts. During the study implementation period, a high-profile case involving an American tourist sparked concern regarding backlash on the Indonesian LGBTQ community in Bali.^12^ Hence, we also took greater care in reaching out to openly gay social media influencers during this time.

However, we believe this would be another important way to disseminate engaging information about the study to the target community. Social media, including via influencers, have played a significant role in promoting education, awareness and access to HIV services amongst gay men in the Philippines, a neighbouring country that is also experiencing a fast-growing HIV epidemic in MSM^13,14^ and similarly in Thailand^15^ and other global settings.^16^ However, the role of using influencers to promote engagement in HIV or global health research has been less described. The use of facilitated film has been utilised to recruit MSM marginalised communities to HIV research in Kenya^17^ a setting where same-sex relationships are criminalised, and LGBT communities also face significant stigma and discrimination. Social interventions that use engaging media and dynamic forms of digital and social media promotion, involving and championed by marginalised communities, can help improve their engagement to health care interventions and research.

Collaborative teams involving diverse community members, creative and social media influencers could facilitate social media engagement across media algorithms and enable a more diverse target population reach. By utilising multi-professional collaborations involving community and creative groups, we believe the potential response from this form of target population engagement, involving short film, interactive website formats and social media, could be amplified to encourage greater involvement of marginalised communities in purposeful research.
